# *Bituminaria antiatlantica* (Psoraleeae, Fabaceae), a new species from Morocco

**DOI:** 10.3897/phytokeys.85.12288

**Published:** 2017-08-31

**Authors:** Salvatore Brullo, Cristian Brullo, Salvatore Cambria, Antonia Cristaudo, Gianpietro Giusso del Galdo

**Affiliations:** 1 Department of Biological, Geological and Environmental Sciences, University of Catania, Via A. Longo 19, Catania 95125, Italy

**Keywords:** Fabaceae, Leguminosae, New species, Endemic, Morocco, Bituminaria, Psoraleeae, Taxonomy, Pollen grain, Seed testa

## Abstract

A new species of *Bituminaria* is described and illustrated: *Bituminaria antiatlantica* Brullo, C. Brullo, Cambria, Cristaudo & Giusso, **sp. nov.**, which is endemic to Anti-Atlas Mountains (Morocco). It is a true chasmophyte, characterized by a suffruticose habit, several woody branches, leaflets coriaceous, rounded to ovate, small, few-flowered inflorescences and corolla pale coloured.

## Introduction

The genus *Bituminaria* Heist. ex Fabricius (Psoraleeae, Fabaceae), is widespread across the Mediterranean region and Macaronesian Islands, where, according to Egan and Crandall (2008), it is estimated to have diverged from other Psoraleoid genera approximately 6.78 million years ago. The first significant diversification of populations in the Psoraleeae occurred about 6.3 mya after the transcontinental split between North America and the Old World (Lavin et al. 2005). Molecular studies have revealed that after this event it radiated rapidly and diversified into many taxa at generic and specific level (Egan and Crandall 2008, Egan and Reveal 2009, Dludlu et al. 2013). Several monophyletic genera have been recognized within the Psoraleeae ((Rydberg 1928, Stirton 1981, 1989, 2005, Grimes 1990, 1997, Egan and Crandall 2008, Egan and Reveal 2009, Dludlu et al. 2013), some of them occurring in the New World (e.g. *Hoita* Rydb., *Orbexilum* Rafin., *Pediomelum* Rydb., *Psoralidium* Rydb., *Rupertia* J. W. Grimes, *Otholobium* C.H. Stirt., *Ladeania* A.N. Egan & Reveal) and other ones in the Old World and Australia (i.e. *Bituminaria*, *Cullen* Medik., *Psoralea* L. and *Otholobium*). Previous taxonomic and phytogeographic investigations have emphasized that several of these genera exhibit an outstanding species richness, which can be explained by evolutionary and ecological processes.

The causes of diversification of the Psoraleeae can be attributed mainly to range fragmentation, geographical isolation, reproductive biology, ecological adaptations, competitive factors, climatic changes and habitat modifications. These speciation processes have been active in the genus *Bituminaria* which is represented by eight distinct species (Stirton 1981, Greuter et al. 1989, Minissale et al. 2013, Giusso et al. 2015, Brullo et al. 2016; Bogdanović et al. 2016): *B. bituminosa* (L.) C.H. Stirt., *B. morisiana* (Pignatti & Metlesics) Greuter, *B. flaccida* (Nábělek) Greuter, *B. basaltica* Miniss., C. Brullo, Brullo, Giusso & Sciandr., *B. kyreniae* Giusso, C. Brullo, Brullo, Cambria & Miniss. (2015: 278), *B. palaestina* (Bassi) Brullo, C. Brullo, Miniss., Salmeri & Giusso and *B. plumosa* (Rchb.) Bogdanović, C. Brullo, Brullo, Ljubičić & Giusso, all belonging to subgenus Bituminaria, and finally *B. acaulis* (Steven ex M. Bieb.) C.H. Stirt. included in the subgenus Christevenia Barneby ex C.H. Stirt.

*Bituminaria* usually colonizes ecologically well-differentiated habitats: *B. morisiana* and *B. kyreniae* are true chasmophytes linked to the Mediterranean climate and both grow on cliffs; *B. flaccida* is exclusively found on sandstone outcrops of desertic areas; *B. palaestina* occurs on moist soils along streams and marshes; *B. basaltica*, *B. bituminosa* and *B. plumosa* grow in steppic grasslands and synanthropic habitats; and finally *B. acaulis* is a mountain species linked to mesophilous open environments within Colchic forests.

Morphologically, the genus Bituminaria subgenus Bituminaria is differentiated by several apomorphic characters, such as determinate capitate inflorescences, as well as pods which are represented by a corpus with a coriaceous pericarp strongly fused with the seed and entirely covered by rigid long white hairs, usually with mixed to black or ivory prickles, very unequal, compact and rigid, while the long beak, inserted in the corpus through a callus, is flat, compact, rigid, provided with very short white and black hairs, forming a dense or scattered indumentum. Other characters shared by all the other species of this subgenus include: trifoliolate petiolate leaves; unequal, entire leaflets, discontinuous, floral vasculature, bracts each subtending 2-3 flowers; calyx gibbose with unequal teeth; corolla anthocyanic; vexillary stamen partially fused with the other filaments; and ovary inserted on a long stalk. Recent taxonomic investigations carried out on *Bituminaria* (Muñoz et al. 2000, Minissale et al. 2013, Bacchetta et al. 2014, Giusso et al. 2015, Toksoy et al. 2015, Brullo et al. 2016; Bogdanović et al. 2016) highlighted that more studies were needed to discriminate clearly the taxa belonging to the *B. bituminosa* complex, which is widely distributed in the Mediterranean territories. As part of these investigations we examined an isolated population of plants occurring in Morocco, which had been described by Maire (1936) as Psoralea bituminosa var. rotundata. To clarify the taxonomic position of this plant, we visited the Anti Atlas Mountains (southern Morocco), during which it was possible to collect specimens used as exsiccata and pods for its cultivation.

Plants referable to Psoralea bituminosa var. rotundata (previously recorded by Maire (1936) and Benabid and Cuzin (1997)) were found in two locations of the subdesertic area. They are represented by Mount Tachilla (*locus classicus*) and Djebel Imzi respectively, where the plant grows in the crevices of cliffs and steep rocky north-facing surfaces. In-depth taxonomic investigations of herbarium specimens and living plants cultivated from wild pods allowed us to observe significant morphological differences between these Moroccan populations and those of other *Bituminaria* species, occurring in various Mediterranean territories. These Moroccan plants (Psoralea bituminosa var. rotundata) are recognised as a distinct species and raised to specific rank as *B. antiatlantica*. The plants are characterized by: a suffruticose habit and very branched, woody stems; coriaceous semi-round to ovate leaves; small, few-flowered inflorescences and pale coloured corollas. It is a rare chasmophyte linked to very dry climatic conditions, and grows together with several other relic endemic species.

## Species treatment

### Bituminaria antiatlantica

Taxon classificationPlantaeFabalesFabaceae

Brullo, C. Brullo, Cambria, Cristaudo & Giusso sp. nov.

urn:lsid:ipni.org:names:77165363-1

Figs 1 , 2



Bituminaria bituminosa *(L.) C.H. Stirt. affinis, sed habitu suffruticoso, ramis lignosis, foliolis glabris vel sparsim pilosis, subrotundatis vel ovatis, max. 35 mm longis, petiolis usque ad 6 cm longis, inflorescentia laxa, saepe subspicata, 1,5-2 cm longa, 3-10-flora, calice 12-13.5 mm longo, corolla pallida*.

**Figure 1.**
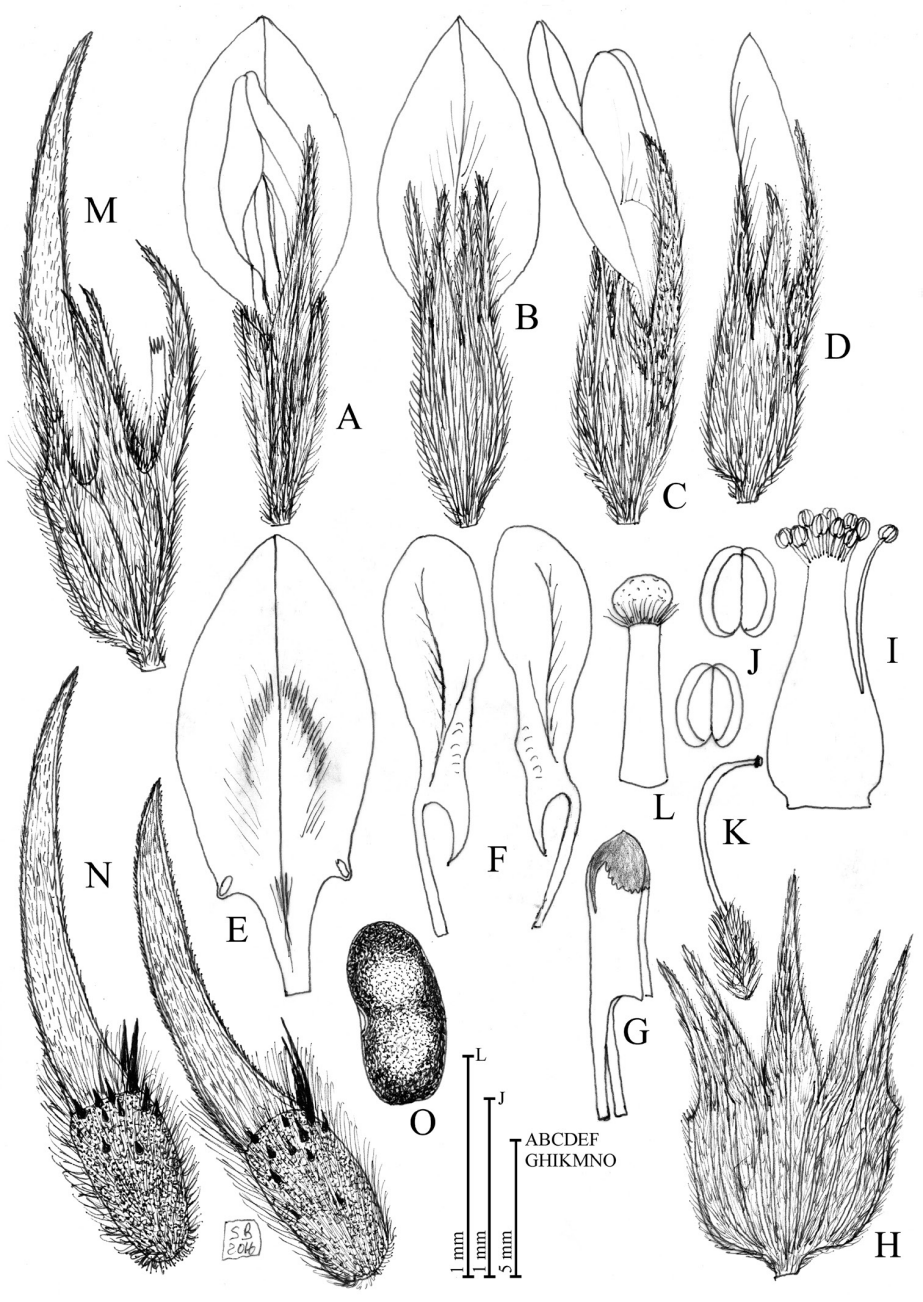
Diagnostic features regarding the reproductive structures of *Bituminaria antiatlantica*. **A** Flower (ventral view) **B** Flower (dorsal view) **C** Flower (lateral view) **D** Bud **E** Standard **F** Wings **G** Keel (lateral view) **H** Calyx (open) **I** Staminal tube **J** Anthers **K** Pisti **L** Stigma **M** Fruiting calyx and pod **N** Pods **O** Seed. Illustration by S. Brullo based on living material coming from Mount Tachilla and Djebel Imzi in Morocco (CAT).

**Figure 2.**
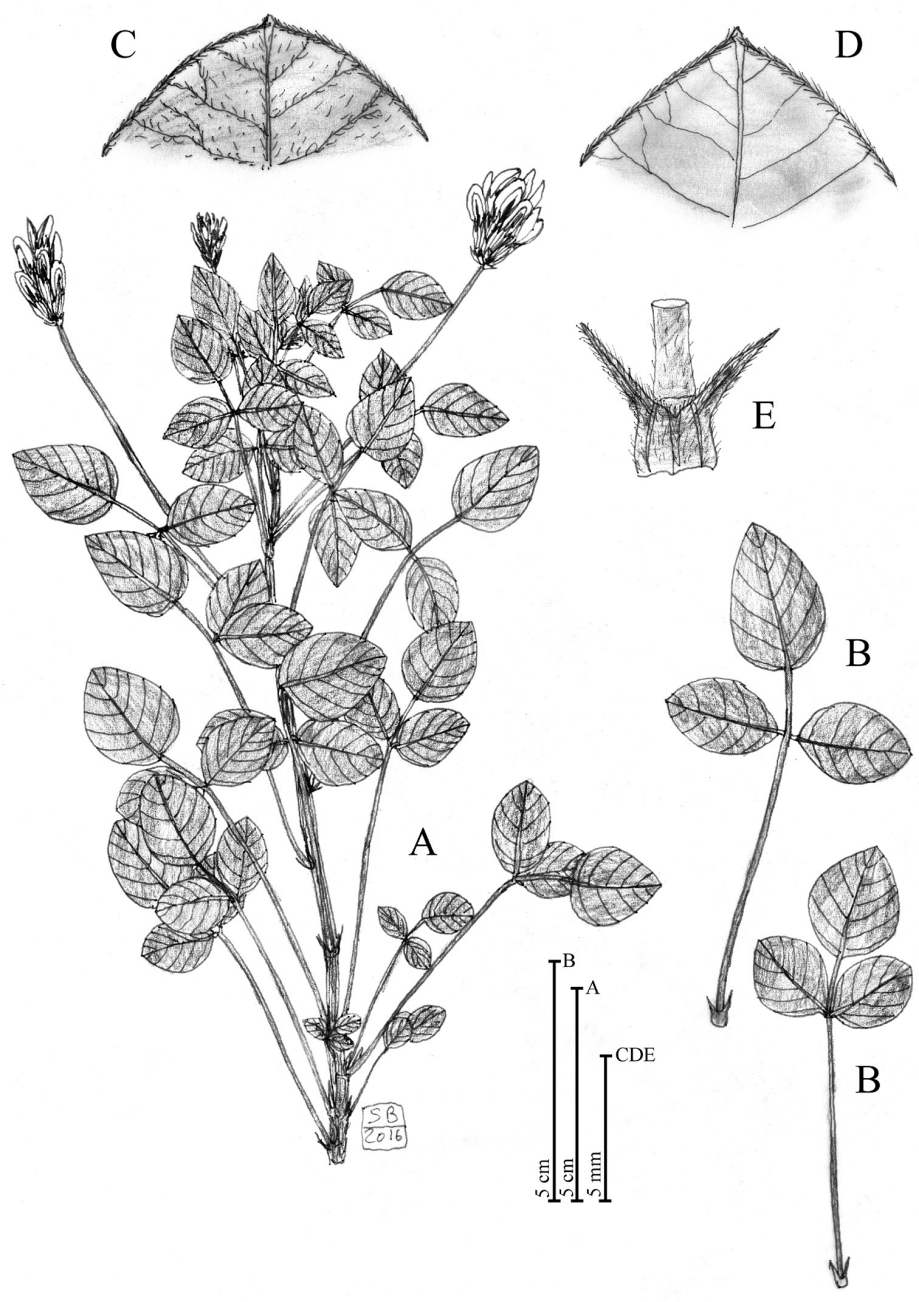
Diagnostic features regarding the vegetative structures of *Bituminaria antiatlantica*. **A** Habit **B** Leaves **C** Leaf apex (abaxial side) **D** Leaf apex (adaxial side) **E** Stipules. Illustration by S. Brullo based on living material coming from Mount Tachilla and Djebel Imzi in Morocco (CAT).

#### Synonym.

Psoralea bituminosa L. var. rotundata Maire, Bull. Soc. Hist. Nat. Afr. N. 27(8): 222, 1936.

#### Type.

Morocco: In rupibus arenaceis Mountis Tachilla ad radices septentr. Anti-Atlantis, 400 m, 10 April 1935, *R. Maire & E. Wilczek s.n.* (holotype MPU!; isotype RAB!), sub Psoralea bituminosa L. var. latifolia Moris f. rotundata).

#### Description.

Perennial, suffruticose, dark green, erect to ascending, up to 60 cm tall. *Stems* dark green-brown, sparsely hairy, with hairs short and appressed, very branched; branches woody, leafy along entire length. *Stipules* 5–6 mm long, rigid, linear-triangular, adnate to the petiole. *Leaves* pinnately 3-foliolate, green, with petiole 1.8–6(7) cm long, sparsely hairy; *leaflets* semi-round to ovate, subglabrous above and sparsely hairy below, 10–35 × 8–21 mm, with apex obtuse to acute, ending in a straight mucro 0.3–0.5 mm long. *Inflorescence* definite, subspicate, lax, 1.5–2 cm long, with 3–10 flowers. *Peduncle* 3.5–14 cm long, overtopping the leaves. *Bracts* 1–3 toothed, 5–8 mm long, subtending 2 or more flowers. *Flowers* 16–17 mm long. *Calyx* 12–13.5 mm long, green, densely hairy, with hairs white mixed to short black hairs; lower teeth 7–8 mm long, laterals shorter, 5.5–7 mm long. *Corolla* whitish-pink to whitish lilac; standard 16–16.5 × 7–7.5 mm, elliptic, striate with lilac in the middle, apex obtuse; wings 14–15 × 3–4 mm; keel 10.5–11 × 2–2.3 mm, having a macula dark violet in the upper part. *Staminal tube* 11–11.5 mm long, with anthers yellow, 0.7–0.8 × 0.3–0.35 mm; vexillary w with filament fused below with the other ones. Pistil 10–10.5 long, ovary hairy, style curved towards the apex, thickened at point of flexure, stigma capitate, penicillate. and ovary hairy. Pod 11–23 mm long (beak included), with beak pubescent, 14–16 mm long. Seed reniform, 6–7 × 3.4–4 mm.

#### Distribution and ecology.

**Figure 3.**
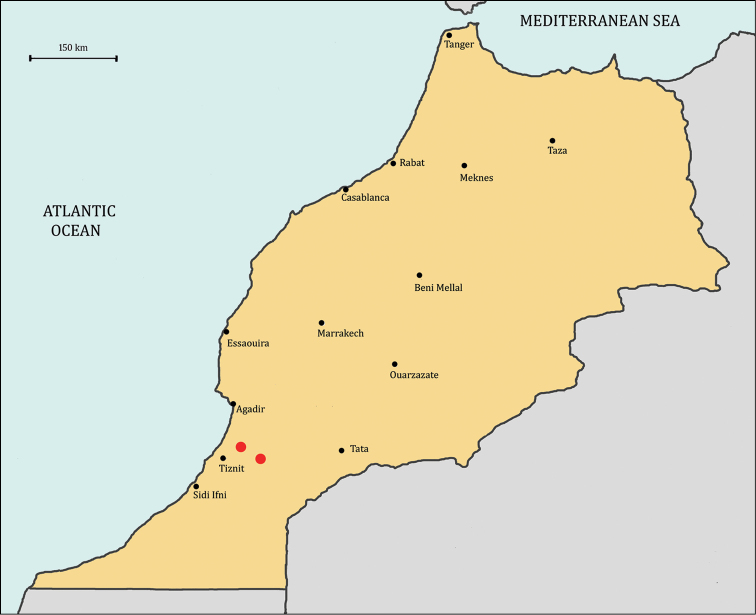
Distribution map of *Bituminaria antiatlantica* (red dots) based on herbarium specimens.

**Figure 4.**
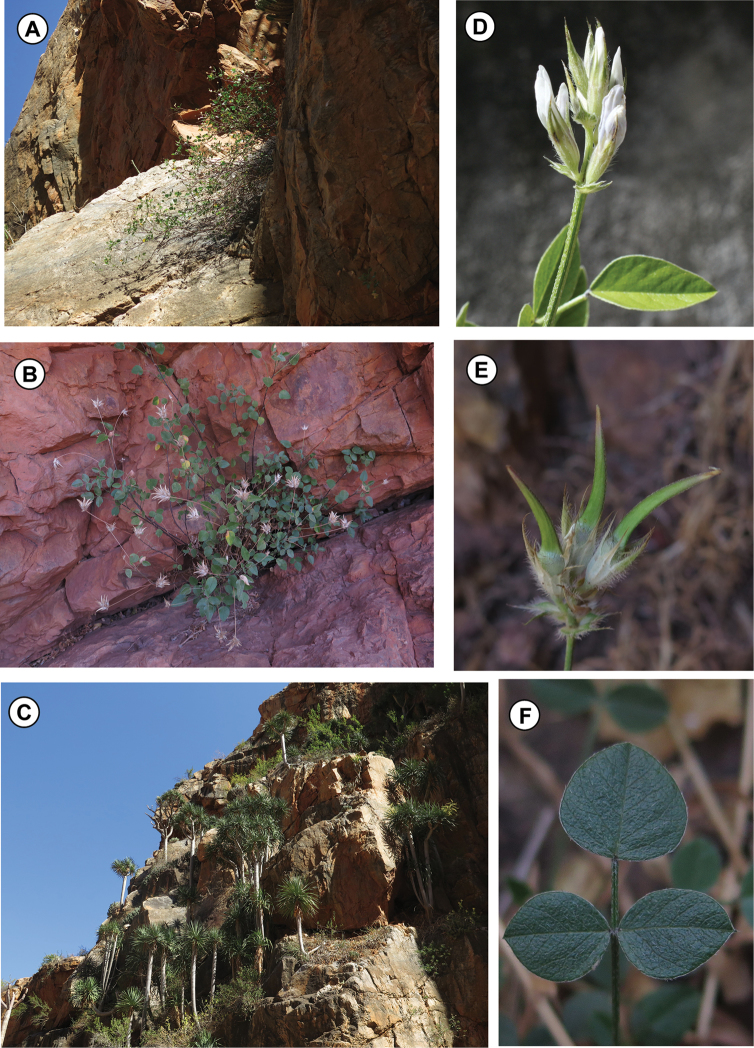
Phenological features of *Bituminaria antiatlantica* **A** Natural habitat with *Bituminaria antiatlantica* in Djebel Imzi (Morocco) **B** Habit of *B. antiatlantica* in Mount Tachilla (Morocco) **C** Natural habitat of *B. antiatlantica* with Dracaena draco L. subsp. ajgal in Djebel Imzi (Morocco) **D** Inflorescence detail of *B. antiatlantica* **E** Fructified inflorescence of *B. antiatlantica* **F** Leaf detail of *B. antiatlantica* (Photos by S. Cambria).

*Bituminaria antiatlantica* is a rare and localized species, currently known only from Mount Tachilla and Djebel Imzi in the Anti-Atlas Mountains in southern Morocco, (Fig. 3). It grows between 300 and 1500 m of altitude, on steep, north-facing slopes, chiefly constituted of Precambrian quartzite. From the bioclimatic viewpoint, this area falls within the infra- and the thermo-mediterranean type, with semiarid to subhumid ombrotype (Benabid and Cuzin, 1997). This species is an element of a chasmophytic vegetation type characterized by Celsia longirostris Murb. var. antiatlantica Emb., *Salvia taraxacifolia* Coss. & Balansa, *Chiladenus hesperius* (Maire & Wilczek) Brullo, *Teucrium werneri* Emb., *Aeonium arboreum* (L.) Webb & Berthel, *Dianthus lusitanus* Brot., *Micromeria hochreutineri* (Briq.) Maire, *Caralluma hesperidum* Maire, *Teline segonnei* (Maire) Raynaud, *Davallia canariensis* (L.) Sm., *Asplenium aethiopicum* (Burm. fil.) Becherer (Fig. 4C). Besides, several phanaerophytes occur in these rupestrian habitats such as Dracaena draco L. subsp. ajgal Benabid & Cuzin, *Laurus azorica* (Seub.) Franco, *Argania spinosa* L., *Kleinia anteuphorbium* (L.) Haw., *Rhus tripartita* (Ucria) Grande, *Euphorbia echinus* Hook.f. & Coss., *Warionia saharae* Benth. et Coss. Many of these species are rare and endemic to the southern part of Morocco, thus highlighting the relic character of this plant community. In particular, Benabid and Cuzin (1997) consider *B. antiatlantica* (sub Psoralea bituminosa var. rotundata) as characteristic of a very peculiar and remarkable association exclusively occurring in a small area of the Anti Atlas range.

#### Etymology.

The specific epithet refers to the Anti-Atlas range, where the species occurs.

#### Conservation status.

Based on current knowledge, *Bituminaria antiatlantica* seems to have a scattered distribution over an area smaller than 2,000 km2. Therefore, following the IUCN criteria (2014), this species should be classified as “Vulnerable” (VU B2). As regards the conservation policy of the growing site, it has been proposed its inclusion in the list of the UNESCO World Heritage Sites for its richness in endemic, rare or important plants, as well as for its breath-taking landscape (see http://whc.unesco.org/en/tentativelists/1180/).

#### Seed and pod micro-morphology.

**Figure 5.**
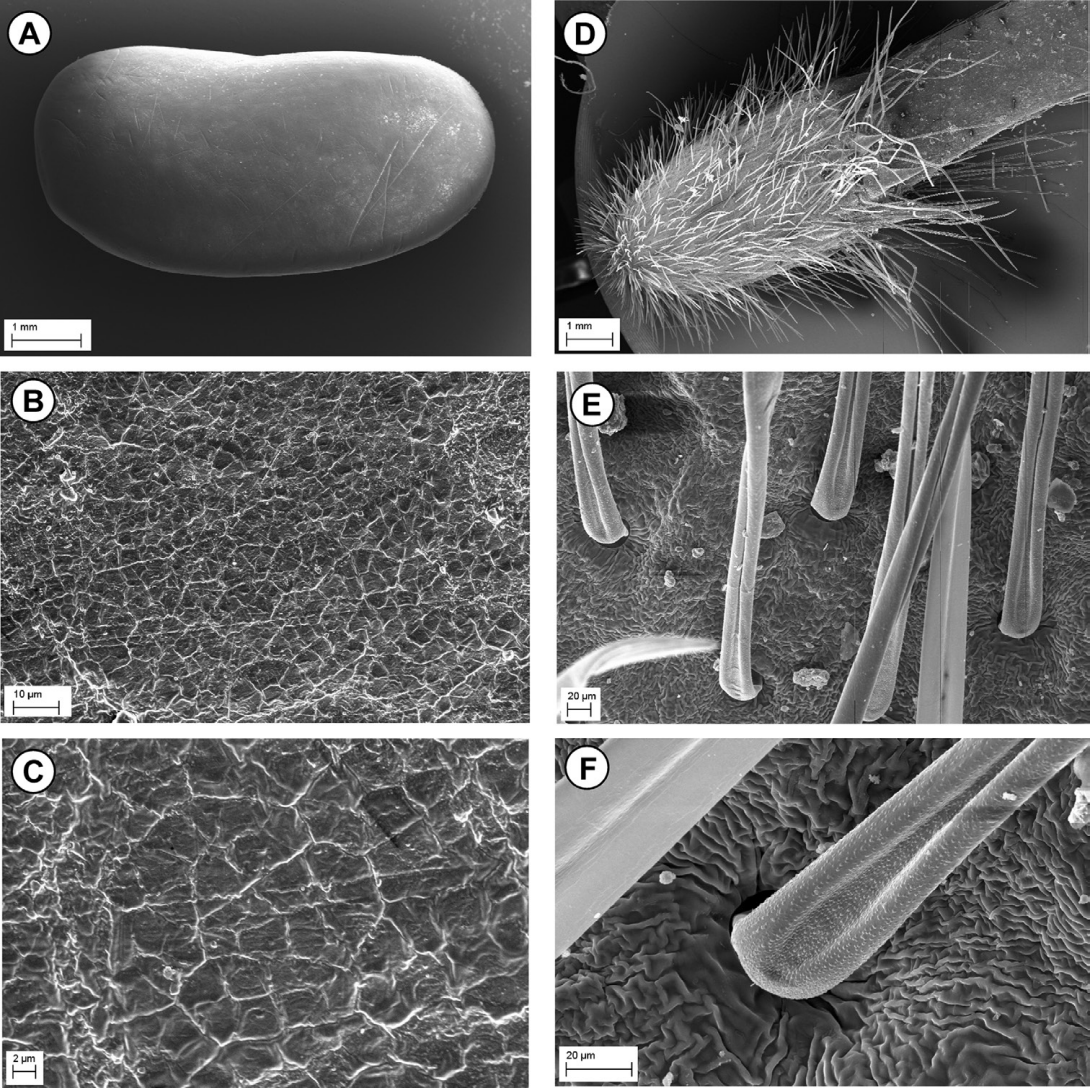
SEM micrographs of seed testa (**A–C**) and pod hairs (**D–F**) of *Bituminaria antiatlantica* from Mount Tachilla in Morocco. **A** Seed at low magnification (× 15) **B** Seed testa at medium magnification (× 1000) **C** Seed testa at high magnification (× 2500) **D** Pods at low magnification (× 10) **E** Pod hairs at medium magnification (× 250) **F** Pod hairs at high magnification (× 700).

As emphasized by several authors (Barthlott 1981, Koul et al. 2000, Kirkbride et al. 2003, Celep et al. 2012, Gandhi et al. 2011 Brullo et al. 2011, 2013) seed coat micro-morphology plays an important role in the taxonomic survey at generic and specific level, especially in those rather critical groups. The seed testa sculptures of *Bituminaria* were investigated by Minissale et al. (2013), Giusso et al. (2015), Brullo et al. (2016) and Bogdanović et al. (2016), who highlighted the systematic relevance of these features providing additional information in order to discriminate among the allied species. In particular, the species hitherto examined are *B. bituminosa*, *B. palaestina*, *B. kyreniae*, *B. basaltica* and *B. plumosa*, which are characterized by different patterns of seed testa. In this study SEM investigations were carried out on the seed of *B. antiatlantica*, according to the protocol of Stork et al. (1980) using the terminology followed Bartholot (1981) and Gontcharova et al. (2009). The results revealed that seed coat sculptures of *B. antiatlantica* differ markedly from the species mentioned above. The seed testa of *B. antiatlantica* is characterized by a fine and inconspicuous reticulum, bordering the cells, which appears irregularly polygonal and 3.5–9(10) µm wide. The anticlinal walls are irregularly curved to straight, slightly grooved and smooth, while the periclinal walls are flat with the epidermis smooth to finely rugose (Fig. 5B–C). The pod corpus is characterized by minutely rugose surfaces, and the indumentum hairs are minutely papillose, with the longitudinal furrow broadly widened at the base (Fig. 5E–F).

#### Pollen morphology.

**Figure 6.**
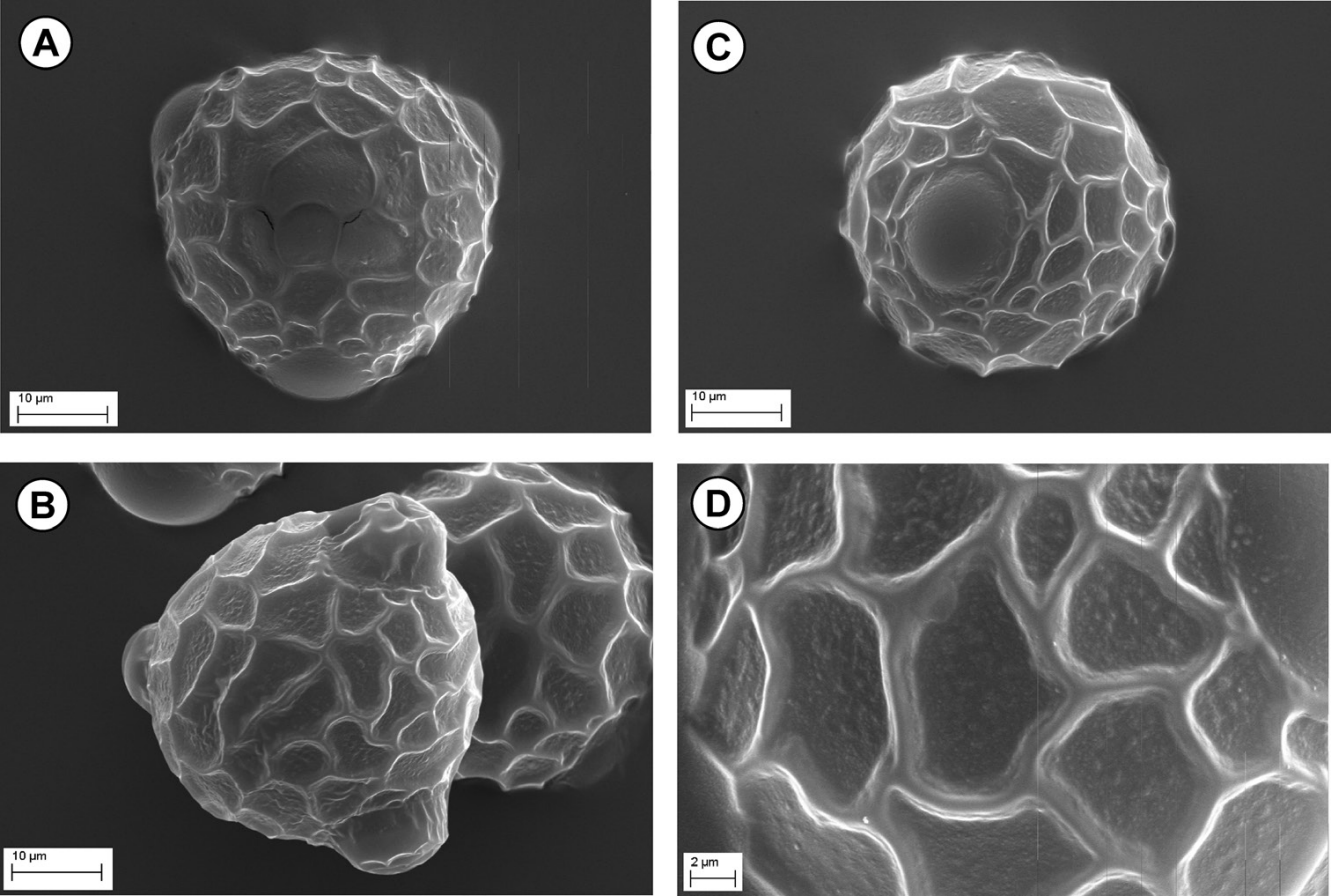
SEM micrographs of pollen grains of *Bituminaria antiatlantica* from Mount Tachilla. **A–B** Polar view (× 1600) **C** Equatorial view (× 1600) **D** Detail of pollen surfaces (× 4000).

Previous studies of pollen morphology of *Bituminaria* included those by La Serna Ramos and Gó Mez Ferreras (2006) and Halbritter and Weis (2015), who published a SEM picture of *B. bituminosa* s.l., while Brullo et al. (2016) examined the pollen grains of *B. bituminosa* s.str. and *B. palaestina*, pointing out distinctive morphological differences between the pollen of the two species. In this study pollen grains of *B. antiatlantica* were excised from flower buds in ahydrated state and were examined according to Walker and Doyle (1975), Punt et al. (1994, 2007) and Hesse et al. (2009). The pollen grains are very similar to that of *B. bituminosa* sensu stricto from Sicily, which has been examined in detail by Brullo et al. (2016), although there are differences in size and ornamentations which distinguishes clearly the pollens of the two species. Actually, The pollen grain of *B. bituminosa* (Brullo et al., 2016, Fig. 6A) is smaller with a size of 25–30(34) µm, with larger brochi (4–17 µm) and fewer in number, showing a laxly papillose lumen and less deep (0.5–1 µm) and narrower (1–1.2 µm) muri. In *B. antiatltantica*, the pollen grain (Fig. 6) is slightly larger (37–38 µm) with smaller brochi (4.3–11 µm) and more numerous, with a lumen minutely papillose and muri deeper (1.4–1.8 µm) and larger (1.4–1.7 µm).

#### Discussion.

*Bituminaria antiatlantica* shares some ecological and morphological characteristics with *B. flaccida*, a very rare species occurring in the semidesert countries of Jordan and Sinai in the Middle East; e.g., reduced leaflets, the size and few-flowered inflorescences, and flower colour. However, the latter differs from *B. antiatlantica* in several significant features (Table 1), such as its herbaceous habit, greyish-glaucous stems and leaves, obovate to linear-lanceolate and densely pubescent cauline leaflets, shorter calyx, longer and slightly retuse standard, shorter staminal tube, and feature of pod and seed.

**Table 1.** Main diacritic features of *Bituminaria antiatlantica* and allied species.

	*B. antiatlantica*	*B. bituminosa*	*B. tunetana*	*B. basaltica*	*B. flaccida*	*B. palaestina*	*B. morisiana*	*B. kyreniae*	*B. plumosa*
Stem habit	erect to ascending	erect (rar. prostrate)	erect to ascending	erect to ascending-erect	erect-ascending	erect	erect-ascending	erect-ascending	erect-ascending
Stem tallness (cm)	up to 60	up to 150	up to 50	up to 60	up to 40	100–200	up to 60	up to 50	up to 150
Stipule length (mm)	5–6	4–15	5–8	3–6	2–7	5–15	8–11	4–10	7–15
Leaf indumentum (abaxial side)	sparsely hair	hirsute	sparsely hairy	hirsute	hirsute	hirsute	sparsely hairy	sparsely hairy	densely villous
Leaf indumentum (adaxial side)	glabrous	hirsute	sparsely hairy	glabrous to subglabrous	hirsute	hirsute	glabrous to subglabrous	glabrous to subglabrous	densely villous
Leaf petiole length (cm)	1.8–6(7)	1.5–15	3–12	4–10	1–7.5	1.5–7	1.5–20	3–12	1.5–6(8)
Basal leaflet shape	rounded to ovate	rounded-elliptical to lanceolate	lanceolate to linear-lanceolate	rounded-elliptical to linear-lanceolate	suborbicular to obovate	widely ovate-subcordate	ovate-lanceolate to elliptical	ovate to lanceolate	ovate
Cauline leaflet shape	rounded to ovate	elliptical to lanceolate	lanceolate to linear-lanceolate	linear	obovate to linear-lanceolate	ovate-lanceolate to lanceolate	ovate-lanceolate to lanceolate	ovate to lanceolate	ovate-lanceolate
Leaflet apex	obtuse to acute	obtuse to retuse	rounded to acute	rounded, apiculate	rounded to acute	obtuse to acute	obtuse to acute	retuse to obtuse	rounded to acute
Leaflet mucro (mm)	0.3–0.5	0.3–0.5	0.5–1.2	0.5–0.8	0.2–1	0.5–1	0.4–0.5	0.3–0.5	1–1.5
Leaflet length (mm)	10–35	3–90	20–70	8–55	4–30	20–55	27–42	12–60	12–65
Leaflet width (mm)	8–21	6–30	5–20	2–15	3–16	14–45	6–20	4–20	6–28
Peduncle raceme length (cm)	3.5–14	8–22	8–14	10–16	14–24	5–12	4–12	5–20	(6)8–21
Raceme shape	subspicate	capitate	subcapitate	capitate	capitate	subspicate	capitate to ovoid	sub-capitate	capitate
Raceme lenght (cm)	1.5–2	2–2.8	1.5–2	1–1.6	1.5–2	2.5–5	2.5–4.5	2–2.8	1.8–3
Raceme (number of flowers)	3–10	15–30	4–12	6–12 (16)	2–8	10–16	10–25	5–10	(10)15–25(30)
Bract length (mm)	5–8	6–15	5–8	6–8	3–5	5–15	6–9	5–12	5–15
Calyx length (mm)	12–13.5	14–18	11–12	10–13	9–12	12–16	15–18	12–16	15–16
Calyx tube length (mm)	5.5–6	6–7	4–5	4–5	5–5.5	(5.5)6–7	5–7	5–8	6–7
Calyx lower tooth length (mm)	7–8	7–12	7–8	6–9	6–7	(5.5)6–7.5(8.5)	7–10	7–11	8–9
Calyx lateral teeth length (mm)	5.5–7	7–9	5.5–7	4–6	5–6	5.5–6(7.5)	6–8	5.5–9	6–7
Corolla (colour)	whitish-pink to whitish-lilac	blue-violet	pinkish-lilac	white	whitish-pink	pale violet	white-violet	blue-violet to violet	purplish-pink
Corolla/calyx ratio	longer	longer	longer	subequalling	longer	longer	longer	longer	longer
Standard shape	elliptical	ovate-elliptical	spathulate	sphatulate	ovate-elliptical	oblanceolate	ovate-lanceolate	oblanceolate-spathulate	elliptical
Standard apex	obtuse	emarginate	slightly retuse	rounded to obtuse	slightly retuse	retuse	obtuse	usually rounded	emarginate
Standard length (mm)	16–16.5	15–20	13–14	11–13	16–19	(17)19–21(24)	18–23	16–24	19–20
Standard width (mm)	7–7.5	5–8	6–7	5–6	7–7.5	7–8(9)	6–8	6–8.5	7–7.5
Wing length (mm)	14–15	14–18	12–12.5	10–11	15.5–16.5	17.5–19	16–18	14–19	18–18.5
Wing limb width (mm)	3–4	2–3	3–3.2	2.5–3	4–4.5	3.4–3.7	3–4	2.8–4	4–4.2
Keel length (mm)	10.5–11	10–14	9–10	7.5–8.5	10–10.5	12–14	11–14	11.5–16	12–13
Keel limb width (mm)	2–2.3	1.8–2.5	1.8–2	1.5–1.8	2–2.2	2.4–2.6	2–2.5	2–2.6	2–2.2
Staminal tube (mm)	11–11.5	10–13.5	8.5–9	7–8	9.5–10	11.5–12.5	9–12	10–15	11–12
Pistil length (mm)	10–10.5	9–12	8–8.5	6–7	9.5–10	12–14	9–10	9–13	13–14
Pod length incl. beak (mm)	21–23	13–26	12–15	9–10	15–16	12–14(16)	18–26	16–22	16–18
Pod beak lenght (mm)	14–16	10–19	9–10	5.5–6	9–10	5–8(10)	12–19	11–17	10–12
Pod beak indumentum	pubescent	pubescent	subglabrous	glabrous	subglabrous	glabrous	pubescent	glabrous	sparsely hairy
Seed lenght (mm)	6–7	5–7	3.5–4.5	3.5–4	5.5–6	6.5–7	5–7	4.5–5.5	5.5–6
Seed width (mm)	3.4–4	3–4	2.8–3	2–2.2	2.7–3	3.7–4.2	3–4	2.4–2.6	4.5–4.8
Ecology	chasmophilous	terricolous xerophilous	terricolous xerophilous	terricolous xerophilous	chasmophilous	terricolous subhygrophilous	chasmophilous	chasmophilous	terricolous

**Other specimens examined (paratypes).** Morocco: Sulle rupi di quarzite arenacea del Jebel Tachilla a circa 200 m di altitudine, 16 June 2015, S. Cambria (CAT!); Sulle rupi di quarzite arenacea in una gola di Jebel Imzi, 300–400 m di altitudine, 19 June 2015, S. Cambria (CAT!); Sulle rupi di quarzite arenacea del Jebel Imzi a 1450 m di altitudine, 18 June 2015, S. Cambria (CAT!).

### Key to the species of Bituminaria subgen. Bituminaria

**Table:** 

1	Cauline leaflets linear; corolla pure white, 11–13 mm long, subequaling the calyx; staminal tube 7–8 mm long, pod (including beak) 9–10 mm long	***B. basaltica***
–	Cauline leaflets wider (not linear), corolla whitish-pink to blue-violet 15–24 mm long, longer than calyx; staminal tube 9–15 mm long, pod (including beak) 12–26 mm long	**2**
2	Raceme 2–10 flowered	**3**
–	Raceme 10–30 flowered	**5**
3	Corolla blue-violet to violet, oblanceolate-spathulate, pod beak thin and soft	***B. kyreniae***
–	Corolla whitish-pink to whitish-lilac, elliptical to ovate-elliptical, pod beak thick and rigid	**4**
4	Stems and leaves greyish-glaucous, hirsute, cauline leaflets obovate to linear, calyx 9–12 mm long, pod (including beak) max 15–16 mm long	***B. flaccida***
–	Stems and leaves dark green, sparsely hair to glabrous, cauline leaflets semi-rotund to ovate, calyx 12–13.5 mm long, pod (including beak) 21–23 mm long	***B. antiatlantica***
5	Stems with patent hairs, basal leaflets widely ovate-subcordate, up to 45 mm wide, raceme mainly in fruit lax and subspicate, pod beak 5–8 (10) mm long	***B. palaestina***
–	Stems with appressed hairs, basal leaflets different shape, max 30 mm wide, raceme always compact and capitate or subcapitate, pod beak 10–19 mm long	**6**
6	Stems and leaves densely villous, leaflet mucro 1–1,5 mm long, corolla purplish-pink, pod beak 10–12 mm	***B. plumosa***
–	Stems and leaves hirsute to subglabrous, leaflet mucro 0.3.0.5 mm long, corolla white-violet to blue-violet, pod beak (10) 12–19 mm long	**7**
7	Leaflets sparsely hairy to glabrous, max 42 mm long; raceme 3–4.5 mm long; corolla white- violet, with standard ovate-lanceolate, obtuse	***B. morisiana***
–	Leaflets hirsute, up to 90 mm long; raceme 2–2.8 mm long; corolla blue-violet, with standard ovate-elliptical, emarginate	***B. bituminosa***

## Supplementary Material

XML Treatment for Bituminaria antiatlantica
